# Bone Marrow Mesenchymal Stem Cells (BM-MSCs) Improve Heart Function in Swine Myocardial Infarction Model through Paracrine Effects

**DOI:** 10.1038/srep28250

**Published:** 2016-06-20

**Authors:** Min Cai, Rui Shen, Lei Song, Minjie Lu, Jianguang Wang, Shihua Zhao, Yue Tang, Xianmin Meng, Zongjin Li, Zuo-Xiang He

**Affiliations:** 1 Department of Nuclear Medicine, State Key Laboratory of Cardiovascular Disease, Fu Wai Hospital, National Center of Cardiovascular Disease, Peking Union Medical College & Chinese Academy of Medical Sciences, Beijing, China.; 2 Department of Radiology, State Key Laboratory of Cardiovascular Disease, Fu Wai Hospital, National Center of Cardiovascular Disease, Peking Union Medical College & Chinese Academy of Medical Sciences, Beijing, China; 3 Department of Nuclear Medicine, Shanxi Provincial People’s Hospital, Taiyuan, China; 4 Department of Pathophysiology, Nankai University School of Medicine, Tianjin, China

## Abstract

Stem cells are promising for the treatment of myocardial infarction (MI) and large animal models should be used to better understand the full spectrum of stem cell actions and preclinical evidences. In this study, bone marrow mesenchymal stem cells (BM-MSCs) were transplanted into swine heart ischemia model. To detect glucose metabolism in global left ventricular myocardium and regional myocardium, combined with assessment of cardiac function, positron emission tomography-computer tomography (PET-CT) and magnetic resonance imaging (MRI) were performed. To study the changes of glucose transporters and glucose metabolism-related enzymes and the signal transduction pathway, RT-PCR, Western-blot, and immunohistochemistry were carried out. Myocardium metabolic evaluation by PET-CT showed that mean signal intensity (MSI) increased in these segments at week 4 compared with that at week 1 after BM-MSCs transplantation. Moreover, MRI demonstrated significant function enhancement in BM-MSCs group. The gene expressions of glucose transporters (GLUT1, GLUT4), glucose metabolism-related enzymes phosphofructokinase (PFK), and glyceraldehyde-3-phosphate dehydrogenase (GAPDH)) and 70-kDa ribosomal protein S6 kinase (p70s6k) in BM-MSCs injected areas were up-regulated at week 4 after BM-MSCs transplantation and this was confirmed by Western-blot and immunohistochemistry. In conclusions, BM-MSCs transplantation could improve cardiac function in swine MI model by activation of mTOR signal transduction pathway.

Acute myocardial infarction (AMI) has seriously shortened human’s life expectancy and lowered the life quality due to its high morbidity and mortality. Although existing treatments such as coronary revascularization could save ischemic myocardium, there are rarely effective treatment measures against necrotic or non-functional myocardium.

Stem cell therapy might ameliorate heart failure by promoting cardiomyocyte regeneration and neovascularization, and recruiting resident stem cells. Bone marrow mesenchymal stem cells (BM-MSCs) are multipotent adult stem cells that reside within the bone marrow microenvironment. Numerous *in vivo* rodent studies have demonstrated the engraftment and differentiation of BM-MSCs within the heart and BM-MSCs have revealed the potency for the treatment of myocardial infarction^2^,^3^.

The therapeutic mechanism of BM-MSCs transplantation has not been fully elucidated. It is considered that these cells possess pluripotent capabilities, including rapid proliferation, induction of angiogenesis, and differentiation into myogenic cells^4^,^5^,^6^. However, more and more studies on BM-MSCs for cardiac therapy questioned the differentiation mechanism^7^. The stem cells’ directional differentiation is a long-term and gradual process, but the transplanted cells in ischemic myocardium cannot survive for a long time. Moreover, low engraftment of transplanted cells could not explain the mechanism of the improved cardiac function^8^. In addition, the number of the survived stem cells settling down is limited in the lesions, but there is often a significant cardiac function improvement, which cannot be sufficiently explained by differentiation mechanisms^9^,^10^.

Previous studies have confirmed that after myocardial ischemia, mRNA and protein expression of enzymes related to glycometabolism played important protective roles in ischemic myocardium^11^. Positron emission tomography (PET) imaging revealed that myocardial 2-Fluorine-18-Fluoro-2-deeoxy-D-glucose (^18^F-FDG) uptake increased after stem cells transplantation^12^, indicating increased glucose metabolism in cardiomyocytes. Moreover, animal experiments showed that in the infarcted border zone energy metabolic abnormalities improved after cells transplantation^13^.

Phosphatidylinositol-3 kinase (PI3K)/protein kinase B (Akt)/mammalian target of rapamycin (mTOR)/eukaryotic translation initiation factor 4E binding protein 1 (4E-BP1)/70-kDa ribosomal protein S6 kinase (p70s6k) signal transduction pathway is a key signal transduction pathway in cell differentiation and cell growth^14^. Arslan *et al*. have shown that MSCs-derived exosomes could increase ATP levels, decrease oxidative stress and activate PI3K/Akt pathway to enhance myocardial viability and prevent adverse remodeling via increasing glycolysis after myocardial ischemia/reperfusion injury^15^. Moreover, previous studies on mTOR signal transduction pathway have revealed positive effects on skeletal muscle regeneration^16^,^17^,^18^,^19^. Considering mTOR signal pathway controls protein synthesis in different levels by increasing the translation of certain mRNAs, we hypothesize here that the activation of mTOR signal transduction pathway by paracrine action of BM-MSCs after myocardial infarction, will further increase cardiomyocyte protein synthesis and glucose metabolism, and then improve systolic function of heart.

## Results

### Animal Mortality

A total of 24 Chinese mini-swine were used for making AMI models, and 20 swine survived. Four animals died before sacrifice (each group 2), which were excluded from further experiment and analysis.

### Assessment of myocardial metabolism by PET/CT

#### Myocardial metabolism assessment of global left ventricle

In the control group, the minimum mean signal intensity (MSI) (MSI in the lowest 18F-FDG uptake segment in left ventricular) increased slightly at 4^th^ week compared with 1^st^ week (37.40 ± 2.28 vs. 35.70 ± 3.02); in the MSCs group, the minimum MSI increased (34.00 ± 4.25 vs. 22.10 ± 3.18). There was a significant difference between the two groups’ increment values, and it was much higher in the MSCs group than that in the control group (11.90 ± 2.93 vs. 1.70 ± 2.00, *P* < 0.05). Similarly, the summed MSI increased in the control group at week 4 compared with that of week 1 (1089.90 ± 24.47 vs. 1084.00 ± 21.15), and in the MSCs group, the summed MSI also increased compared with 1^st^ week (1075.50 ± 28.30 vs. 1013.50 ± 29.37). The MSCs group’s increment was much higher compared with the control group (62.00 ± 23.30 vs. 5.90 ± 27.98, *P* < 0.01). However, other indexes such as SRS, SRS%, LV defect area (Defect, cm^2^), defect area extent (Extent, %), total perfusion defect (TPD, %), etc, had no significant differences between the two groups (Suppl. Table 2).

#### Regional myocardial metabolism assessment of left ventricle

**Figure 1:**
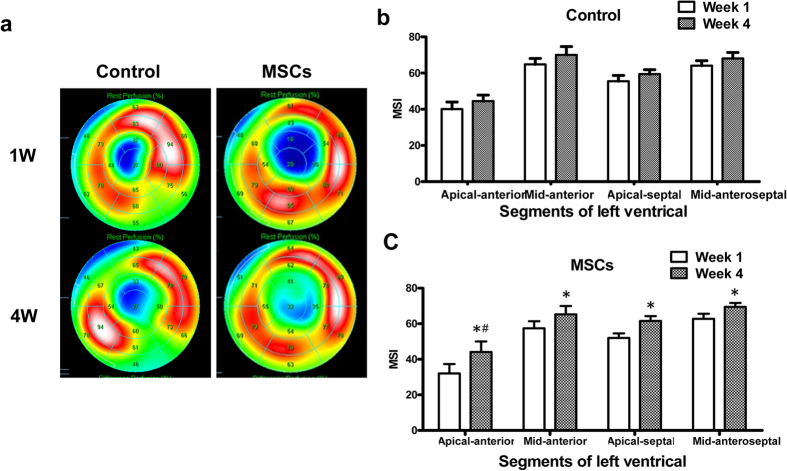
^18^F-FDG PET imaging of cardiac viability. (**a**) Representative polar map of the PET images obtained from pigs treated with PBS versus MSCs at week 1 and week 4. (**b,c**) Myocardial metabolism evaluation by measuring the left ventricular segmental average signal intensity (mean signal intensity, MSI) at Control group (**b**) and MSCs group (**c**). *MSI increased significantly, *P* < 0.05; ^#^MSI increment value was significantly higher in the MSCs group than in the control group, *P* < 0.05.

**Figure 2:**
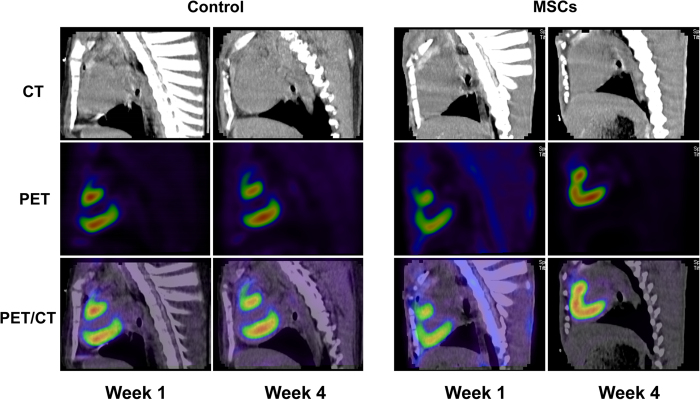
Representative PET-CT images at week 1 and 4 in infarcted hearts receiving PBS versus MSCs. In the MSCs group, the myocardial infarct area decreased at week 4 compared with week 1.

Metabolic evaluation in regional left ventricle showed that in the control group at week 4, the apical-anterior segment MSI was not significantly affected compared with week 1 (44.50 ± 3.36 vs. 40.10 ± 3.94); While in the MSCs group, the apical-anterior segment MSI increased significantly from week 1 to week 4 (32.00 ± 5.35 vs. 44.10 ± 5.90). Moreover, MSI increment value of the apical-anterior segment was much higher than that of control group (14.80 ± 4.10 vs. 4.60 ± 4.98, *P* < 0.05). In the mid-anterior segment, apical-septal segment and mid-anteroseptal segment MSI, in both groups, was increased at week 4 compared with that of week 1, but the increment values between the two groups had no statistical significance (Figs 1 and 2, Suppl. Table 3).

### Cardiac function evaluation by myocardial MRI

**Figure 3:**
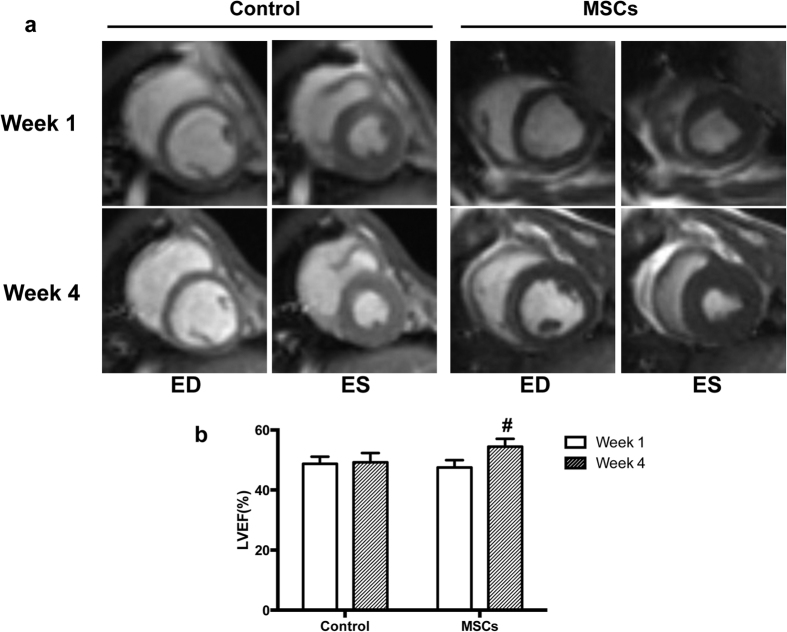
Cardiac function evaluation by MRI. (**a**) Representative MRI image. In the MSCs group, LVEF was significantly increased at the 4^th^ week compared with the 1^st^ week, and ESV was significantly decreased. However, there were no significant differences in the control group. (**b**) Histogram shows cardiac function changes between the 1^st^ and 4^th^ week in the two groups. LVEF improved significantly in the MSCs treated group. ^#^*P* < 0.01.

MRI examination was performed to evaluate cardiac function. LVEF was significantly increased at week 4 compared with that of week 1 in the MSCs group (54.41 ± 2.62 vs. 47.54 ± 2.43), and the increment value was significantly higher than that of the control group (6.87 ± 1.48 vs. 0.47 ± 2.13, *P* < 0.05) (Fig. 3). ESV, at week 4, significantly decreased in the MSCs group (22.85±1.91 vs. 27.07 ± 1.67), and the difference values in the two groups were significantly different (1.06 ± 1.96 vs. −4.22 ± 0.97, *P* < 0.01) (Suppl. Table 4).

### Gene and protein expression analysis

**Figure 4:**
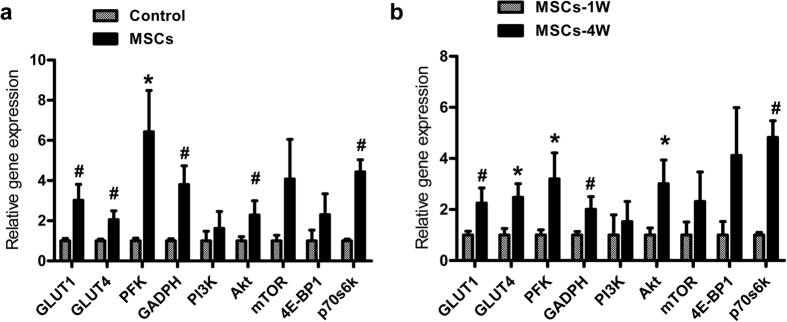
qPCR analysis revealed that MSCs transplantation could increase glucose metabolism and mTOR pathway genes expression. (**a**) The mRNA expression of these genes were increased in MSCs treated group compared with PBS group at week 4. (**b**) Moreover, these genes were higher expressed in MSCs group at week 4 than of week 1. ^#^*P* < 0.01, ^*^P < 0.05.

To evaluate the cell viability after the BM-MSCs transplantation, we performed histology analysis of cardiac tissue sections from the BM-MSCs injection regions. Representative photographs of DAPI-positive nuclei of transplanted BM-MSCs at week 1 and week 4 are shown in Suppl. Fig. 4. DAPI staining in present study confirmed rapid cell loss after transplantation (Suppl. Fig. 4). Thus, we believe that the therapeutic effects of stem cell transplantation should be mostly attributed to the paracrine effects. qPCR results demonstrated that the levels of GLUT1, GLUT4, PFK and GAPDH in the MSCs group were significantly higher than those in the control group at week 4 (^*^P < 0.01, ^#^*P* < 0.05; Fig. 4a). Compared with week 1, the mRNA expression of the genes mentioned above in the MSCs group were also significantly increased at week 4 (^*^P < 0.01, ^#^*P* < 0.05; Fig. 4b). mTOR signal transduction pathway related genes, Akt and p70s6k gene expression levels were higher in BM-MSCs group at week 4 than that of week 1. Moreover, Akt and p70s6k gene expressions were increased in BM-MSCs groups compared with control group at week 4 (^*^P < 0.01, ^#^*P* < 0.05). PI3K, mTOR and 4E-BP1 were higher in BM-MSCs group at week 4, but no significant difference (*P* > 0.05) (Fig. 4).

**Figure 5:**
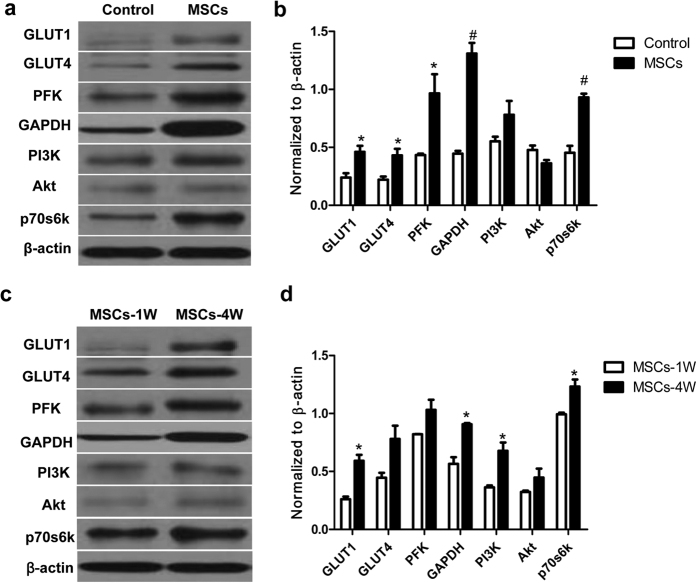
Western blot analysis revealed that MSCs transplantation could increase glucose metabolism and mTOR pathway genes expression. (**a**) These proteins were increased in MSCs treated group compared with PBS group at week 4. (**b**) Moreover, these proteins were higher expressed in MSCs group at week 4 than week 1.

To further investigate the protein level changes of these significantly up-regulated genes, including GLUT1, GLUT4, PFK, GAPDH, Akt and p70s6k, we analyzed these proteins and PI3K by Western blot. The expression of these proteins were up-regulated in BM-MSCs group at week 4 either compared with control group at week 4 or with the BM-MSCs group at week 1 (Fig. 5).

**Figure 6:**
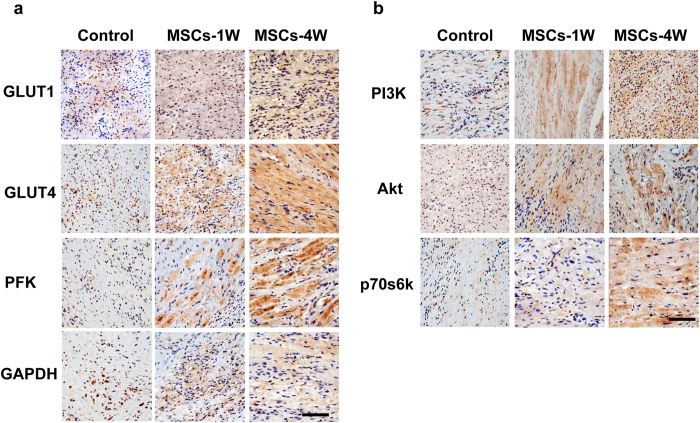
Immunohistochemical staining of cardiac tissue from MSCs injection regions. The positive protein expressions could be seen in the MSCs transplanted groups (either at week 1 or week 4) versus the control group. Moreover, the intensity of immunohistochemical staining was significantly higher at week 4 than week 1 in the MSCs group. (**a**) The proteins include GLUT1, GLUT4, PFK and GAPDH. (**b**) The mTOR pathway proteins include PI3K, Akt and p70s6k. Scale bar = 100 μm.

Histopathological study of cardiac tissue sections from the BM-MSCs injection regions was performed to determine morphological changes. Positive expression was observed for all examined proteins (GLUT1, GLUT4, PFK, GAPDH, PI3K, Akt and p70s6k) which could be seen in the BM-MSCs transplanted groups versus the control group. Furthermore, the intensity of immunohistochemical staining was significantly higher in the BM-MSCs group at week 4 than that of BM-MSCs group at week 1 (Fig. 6a,b, ×400).

## Discussion

**Figure 7:**
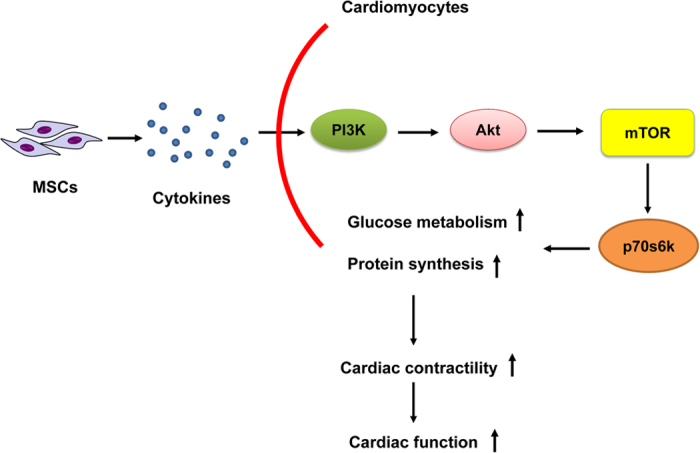
Schematic diagram of the mechanism of cardiac function enhancement of BM-MSCs for the treatment of ischemic heart disease. PI3K/Akt/mTOR has been considered as a central regulatory pathway of protein translation in the regulation of cell proliferation, growth, differentiation, migration and survival. During hormonal stimulation, the mTOR can be activated through phosphorylation by upstream pathways, such as PI3K and Akt. After BM-MSCs transplantation, MSCs secrete cytokines to activate PI3K/Akt/mTOR pathway, and as downstream effective molecule, mTOR specifically phosphorylates ribosomal protein p70s6k, and then p70s6k promote myocardial glucose metabolism, protein synthesis and then to improve cardiac function.

The results of this study revealed that intramyocardial transplantation of BM-MSCs after AMI could improve cardiomyocytes’ glucose metabolism and cardiac function. The gene expressions, including glucose transporters (GLUT1, GLUT4), glucose metabolism-related enzymes (PFK, GAPDH), Akt and p70s6k, were up-regulated at week 4 after BM-MSCs transplantation in BM-MSCs injection areas. We speculate that BM-MSCs may simultaneously activate certain signal transduction pathways through secretion of certain cytokines, thus promote myocardial glucose metabolism and ATP production, and then improve cardiac function (Fig. 7). Our results indicate that BM-MSCs may activate mTOR signal transduction pathway through paracrine which in turn promote myocardial glucose metabolism and cardiomyocytes’ regeneration, and then give rise to improved myocardial contraction and enhanced cardiac function.

### Stem cell therapy for cardiovascular diseases

Studies in animal models of myocardial infarction have demonstrated the ability of transplanted MSCs to engraft and differentiate into cardiomyocytes and vasculature cells, recruit endogenous cardiac stem cells, and secrete a wide array of paracrine factors^3^,^20^. In 2001, Orlic *et al*. reported that intramyocardial injection of undifferentiated lineage-negative BM-MSCs in a mouse model of MI resulted in the formation of new cardiac tissue for the first time^21^. However, some studies in animal models have also challenged the direct differentiation mechanism that the exogenous populations regenerate into cardiovascular components^4^,^22^,^23^,^24^. In fact, while engraftment and differentiation of administered cells have been demonstrated to be possible, these events are rare and occur at very low levels. Nevertheless, studies have consistently demonstrated improvements in cardiac function and ventricular remodeling following MSC therapy, providing evidence that paracrine-mediated effects may be the driving mechanism in cardiac regeneration.

The swine with its large heart/body weight and similarities to the human cardiovascular system is a valuable pre-clinical animal model for stem cell therapy^25^. Our previous research has confirmed that bone marrow-derived mononuclear cells’ coronary delivery may improve cardiac function and positive ventricular remodeling in the heart with AMI^26^. These results demonstrated that functional and metabolic regeneration of infarcted tissue could be realized in humans by bone marrow mononuclear cell transplantation^27^. Similarly, in the TOPCARE-AMI study^28^, 26 patients underwent bone marrow-derived cells transplantation after AMI, and first observations of the effect of local progenitor cell infusion on the regeneration of infarcted cardiac tissue after AMI was evaluated by means of ^18^F-FDG PET and ^201^Tl single photon emission computed tomography (SPECT). In this study, our results revealed robust cell surviving at week 1 after MSC transplantation. However, this population became significantly decreased when the tissues were examined at week 4. These findings indicate that other mechanisms such as activation of paracrine pathways may play an important role on cardiac performance amelioration at week 4. Moreover, our bioluminescence imaging (BLI) data on transplantation of endothelial cells for heart ischemia therapy revealed only 1.5–2.0% survival after 4–8 weeks^1^,^29^,^30^. It has been widely agreed that the most critical mechanism by which stem cells confer reparative benefits are their paracrine actions including anti-inflammatory, pro-proliferative, anti-apoptotic, and pro-angigenic effects^31^. Considering the therapeutic efficacy of MSCs is associated with cell engraftment efficiency, strategies aimed at prolonging cell survival with biomaterials to extend paracrine activation should be introduced for future stem cell therapy^10^,^32^,^33^.

### Increased glucose metabolism and cardiac function after MSCs transplantation

In our study, ^18^F-FDG’s uptake increased significantly in the injection areas and even the in global left ventricular at week 4 compared with week 1 after MSCs transplantation, with cardiac function improved and ESV reduced. These experimental results show that the improvement of myocardial systolic function might be associated with glucose metabolism increasing, which are consistent with those results from the randomized controlled trials mentioned above.

Glucose, which cannot freely pass through the lipid bilayer of cell membrane, is taken into cells via GLUTs on the cell membrane. The protein family of GLUTs comprises 14 isoforms that share common structural features such as 12 transmembrane domains, N- and C-termini facing the cytoplasm of the cell, and a N-glycosylation side either within the first or fifth extracellular loop^34^. GLUT1, a housekeeper protein, plays an important role in mediating myocardium glucose uptake in the basal metabolic state, and GLUT4 is associated with the maintenance of cardiac function^35^,^36^,^37^.

Mammals’ phosphofructokinase (PFK), the main rate-limiting enzyme in glycolysis, is a major adjustment point in this process^38^, during which PFK phosphorylates fructose-6-phosphate into fructose-1,6-diphosphate. Glyceraldehyde-3-phosphate dehydrogenase (GAPDH) is another classic enzyme participating in glycolysis, which dehydrogenates glyceraldehyde-3-phosphate into 1,3-diphosphoglycerate. At present, few researches focus on changes of the glucose related enzymes in local myocardial tissue after MSCs transplantation. Our research showed that, at 4 weeks after injection of MSCs, PFK and GAPDH gene expression were significantly increased in injection areas of myocardial tissue compared with control group, and the expression of these genes and proteins were increased at week 4 compared with week 1 after MSCs transplantation. These elevated expressions were related with increased tendency of myocardial glucose metabolism detected by ^18^F-FDG PET. It was showed that at week 4, both the GLUTs as the glucose metabolic enzymes kept rising. Considering the limited residence time of transplanted stem cells, it is speculated that MSCs may secret certain cytokines which activate special signaling pathways, which induce signal cascade amplification resulting in persistent enzyme activities in the cells.

### Increased expression of genes and proteins of mTOR/p70s6k in MSCs transplanted areas

PI3K/Akt/mTOR has been considered as a central regulatory pathway of protein translation in the regulation of cell proliferation, growth, differentiation, migration and survival^39^,^40^. Most studies have confirmed that the growth of skeletal muscle cells associates with mTOR pathway^17^,^18^, and mTOR plays an important role in the processes of cardiac hypertrophy and myocardial fibrosis^41^. In a rat model of myocardial hypertrophy (after ligation of arteries), the activity of p70s6k (mTOR’s downstream signaling molecules) increased significantly. Treatment with rapamycin (mTOR inhibitor) significantly inhibited the activity of p70s6k and myocardial hypertrophy was alleviated^42^. These results indicated that mTOR was a key regulator in glucose and lipid metabolism, and rapamycin was shown to modulate glucose transport, preventing long-term insulin-induced increases in GLUT1 protein synthesis via partial inhibition at the level of both transcription and translation^42^. A study has demonstrated inhibition of the mTOR/p70s6k1 pathway mainly by blunting GLUT4 expression^43^. Recently, Pereira *et al*. pointed out that mTOR was the central regulator of glucose metabolism in human adipocytes, in which rapamycin reduced glucose uptake through impaired insulin signaling^44^. However, few researches focus on the effect of mTOR on glucose metabolism in cardiac myocytes. In our study, myocytes’ glucose metabolism was increased accompanied with enhancement of GLUTs and myocardial metabolic enzymes expression. In the local area of MSCs injection, the expression of mTOR had an increasing tendency but no statistical significance. We assumed that after MSCs transplantation, MSCs secrete cytokines to activate Akt/mTOR/p70s6k pathway, and as downstream effective molecule, p70s6k promoted myocardial glucose metabolism.

### Limitations

In this study, the open-chest surgery which we used to make infarction model is apparently an invasive approach, and the healing wounds in chest wall could affect cardiac glucose metabolism imaging on PET; however, less invasive methods (e.g., intracoronary catheter-based procedures) might be technically difficult for accurately injecting stem cells. Additionally, the corresponding channel inhibitors were not used in this mTOR signaling pathway study. If myocardial glucose metabolism and cardiac function obviously decrease after rapamycin treatment, it will be an additional proof that mTOR pathway is activated by MSCs transplanted in infracted myocardium. Therefore, the next step in our research will include addition of mTOR pathway inhibitors to further confirm our current research findings.

## Conclusions

The present study demonstrated that intramyocardial injection of BM-MSCs can promote myocardial glucose metabolism and improve cardiac function post AMI. In the injection region of MSCs, expression of GLUTs and glycolytic enzymes are up-regulated, associated with increased glucose metabolism. The increased expression of mTOR/Akt/p70s6k probably indicated that BM-MSCs may activate this pathway via paracrine actions, then promote myocardial glucose metabolism and cell growth, and further improve cardiac contractility and cardiac function.

## Materials and Methods

### Animals

Twenty-four ten-month-old Chinese mini-swine (25 ± 5 kg) were obtained from the Laboratorial Animal Center of the Chinese University of Agriculture, China. All animals received humane care in compliance with the *Guide for the Care and Use of Laboratory Animals* published by the National Institutes of Health, USA. Experimental procedures were approved by the Care of Experimental Animals Committee of the Chinese Academy of Medical Sciences and the Peking Union Medical College, China. The animals (n = 24) were randomly divided into BM-MSCs Group (n = 12) and Control Group (n = 12).

### Isolation, culture and labeling of MSCs

Swine were sedated with ketamine (Gutian Company, Fujian, China, 35 mg/kg intramuscular), induced with valium (Jinyao Company, Tianjin, China, 1.5 mg/kg), and maintained anesthetized through the intravascular injection of ketamine and valium. The left iliac crest area was prepared and about 25 ml of bone marrow was aspirated with a bone marrow aspiration needle into a syringe containing 12,500 units of heparin.

BM-MSCs isolation and culture for autologous transplantation were performed as previously described with some modifications^45^. Briefly, the bone marrow aspirates were doubly diluted with phosphate buffer saline (PBS) and then mononuclear cells were isolated by centrifugation through 1.077 g/ml Percoll (Sigma, USA) at 800 × *g* and 4 °C for 30 minutes. The mononuclear cells were then rinsed twice with PBS at 400 × *g* and 4 °C for 10 minutes and plated at a density of 5 × 10^5^/cm^2^ in Iscove’s Modified Dulbecco’s Medium (IMDM, Gibco, USA), with 10% fetal bovine serum (Gibco), 100 U/ml penicillin and 100 μg/ml streptomycin (Gibco). The medium was changed every 3 days. After about 10 days in culture, the adherent cells formed homogeneous gyrate colonies. When the cells reached 80% confluence, they were detached using 0.25% trypsin-EDTA (Invitrogen) and subcultured at the ratio of 1:3. The phenotype of BM-MSCs was analyzed using the following antibodies, phycoerythrin (PE)-conjugated CD44, CD34, and fluorescein isothiocyanate (FITC)-conjugated CD90, CD45, HLA-DR. PE or FITC isotype-matched antibodies served as controls. Cells were examined by LSRII flow cytometer (BD Bioscience, San Jose, CA, USA).

At 80% confluence, the cells were detached and re-suspended in a tube containing IMDM without fetal bovine serum and labeled with 4′,6-diamidino-2-phenylindole (DAPI; Sigma, USA) for 30 minutes at 37 °C. The cells were rinsed three times with PBS to remove unbound DAPI and kept in warm IMDM (3 × 10^7^−4 × 10^7^ cells per swine) for a few minutes before transplantation. This labeling procedure was very efficient, ensuring almost 100% labeling of cell nuclei.

### AMI model and cell transplantation

The mini-swine were sedated again, endotracheally intubated, connected to a ventilator (Narkomed, Germany) and anesthesia was maintained through intravascular injection of ketamine and valium. The animals were intubated with a cuffed endotracheal tube and ventilated with 100% oxygen to maintain arterial carbon dioxide tension (PaCO_2_) between 35 and 45 mmHg. Electrocardiography was used to monitor heart rate, rhythm, and ST-segment changes during the surgical procedure.

A midline sternotomy was performed and the left anterior descending (LAD) coronary artery was dissected free just distal to the first diagonal branch and isolated with a vessel loop. A 90-minute occlusion of the LAD was used to produce AMI. Lidocaine infusion (Hualu Company, Shandong, China, 2 mg/kg i.v. bolus, and then 0.5 mg/min i.v.) was started and continued. Thirty minutes after occlusion of the LAD, 1 ml cell suspensions of autologous BM-MSCs (3 × 10^7^ cells per swine) were intramyocardially injected into the left ventricular wall of the peri-infarct zones (200 μl into 5 foci) with a 28-gauge needle. The animals in the control group received intramyocardial injection of 1 ml PBS.

At the end of transplantation, the swine chest was closed. The anesthesia was then stopped; the animal was extubated when appropriate and allowed to recover. Animals were treated postoperatively with an antibiotic (cephazoline, 1.0 gram intramuscularly, twice daily for 3 days) and an analgesic (buprenorphine, 0.3 mg intramuscularly, twice daily for 3 days).

### ^18^F-FDG PET cardiac glucose metabolic imaging

One week (baseline) and four weeks (endpoint) after MSCs transplantation, F-18-fluorodeoxyglucose–positron emission tomography (FDG-PET) was performed with a cardiac PET scanner (TruePoint™, Siemens, Germany). The animals were anesthetized through the intravascular injection of ketamine and valium, and then eight IU insulin was injected intravenously. Twenty minutes later, at the time of decrease of glucose level, 2–3 mCi ^18^F-FDG was administered, and 1 hour after administration of FDG, acquisition started. Standardized quantitative analysis was performed with FDG-PET bull’s-eye views and calculating the mean signal intensity (MSI) in the respective areas based on a 17-segment model^46^.

The imaging data were analyzed by QGS^TM^ software (version 3.1, Cedars-Sinai Medical Center, Los Angeles, California, USA). Manual fitting was applied when the mitral valve plane or left ventricle contour was inappropriate for visual interpretation. The results of the analysis were obtained using the American Heart Association (AHA) and segmental scoring method standard solutions^28^, measuring the left ventricular segmental average signal intensity (mean signal intensity, MSI). The value of segment with max MSI was set as 100%, the values of other segments were counted automatically according to the max MSI. The metabolism is suggested to be decreased in the segments with MSI < 70%. For the assessment of regional metabolism, one experienced nuclear medicine physician assessed PET images visually. Summed rest score (SRS), summed rest score percentage (SRS%), perfusion defect area (Defect, cm^2^), perfusion defect extent (Extent, %) and total perfusion defect (TPD) were obtained by QPS software, and summed difference score (SDS, SRS_1W_−SRS_4W_) and SDS% (SRS%_1W_−SRS%_4W_) were calculated separately by adding the scores of the 17 segments (Supplemental Figs 2 and 3). All above indexes reflect left ventricular myocardial glucose metabolism.

### Heart function assessment by MRI

All the experimental animals were studied at both baseline and endpoint by cine MRI and contrast-enhancement MRI. MRI was performed using a 1.5 T clinical MRI scanner (Siemens Avanto, Germany) with a phase-array radiofrequency receiver coil. The MRI was wireless vector electrocardiogram gated. Both the cines and the corresponding contrast enhancement MRI were prescribed every 4 mm from the base to the apex in the LV short-axis images, starting at the level of the mitral valve, which resulted in 6 to 8 short-axis slices. Cine MRI images were acquired using a True fast imaging with steady precession (FISP) sequence with a time-adaptive sensitivity encoding technique. Typical imaging parameters were as follows: repetition time, 41.7 ms; echo time, 1.39 ms; bandwidth, 965 Hz/Pz; flip angle, 48 degrees; image matrix, 109 × 192; in-plane resolution, 3.2 × 2.0 mm; and slice thickness, 6.0 mm. Contrast enhancement MRI was performed directly 10 minutes after the administration of 0.2 mmol/kg of Gd-DTPA (Magnevist, Schering). T1-weighting was achieved using a phase sensitivity inversion recovery (PSIR) fast low-angle shot sequence. Time of inversion was auto adjusted by the PSIR technique. Typical image parameters were repetition time (700 ms); echo time (4.8 ms); bandwidth (130 Hz/Pz); in-plane resolution (1.8 × 1.3 mm); image matrix, 256 × 256; and slice thickness (8 mm).

All the MR images were quantitatively analyzed by commercial software provided by Siemens (Syngo VD10B, Syngo VX49B, Argus VA60C, Siemens AG, Medical Solutions, Erlangen, Germany). Cardiac function parameters were analyzed, including left ventricular ejection fraction (LVEF), left ventricular end-diastolic volume (LVEDV), left ventricular end-systole volume (LVESV), left ventricular stroke volume (LVSV), left ventricular cardiac output (LVCO), left ventricular cardiac index (LVCI), left ventricular mass end diastolic (LVMASS-ED).

### Tissue harvesting

At the end of experiments, swine were given euthanasia and the myocardial tissue samples in the size of 10 mm × 10 mm × 10 mm were obtained in the injection points with MSCs or PBS. Two tissue samples were snap frozen in liquid nitrogen and preserved in −80 °C refrigerator for Real-time Quantitative PCR (qPCR) and Western-Blot. One sample was fixed in 4% paraformaldehyde for 24 hours, embedded in paraffin and sectioned into 5-μm slices for immunohistochemistry.

### qPCR assay

Real-time polymerase chain reaction (PCR) was performed for quantification of the glucose transporters (GLUT1 and GLUT4), glucose metabolism enzymes (phosphofructokinase, PFK; glyceraldehyde 3-phosphate dehydro genase, GAPDH), and PI3K/Akt/mTOR/4E-BP1/p70s6k gene expression after cell transplantation. The swine myocardium tissue samples from different groups were frozen in liquid nitrogen and powdered. The primer sequences of analyzed genes were shown in Suppl. Table 1. qPCR was performed on the Bio-Rad CFX96 real-time PCR thermal cycle instrument (Applied Biosystems, Hercules, Cal, USA) using UltraSYBR Mixture (CWbio.Co.Ltd, *Cat#CW0956*, China). Gene expression level was quantitated relative to the expression of the reference gene (β-actin) by employing the 2^−ΔΔCT^ value models^47^. The expression data were calculated using the Bio-Rad CFX Manager, version 2.1 software (Bio-Rad Inc., Hercules, CA, USA).

### Western-Blot assay

To investigate the effects of MSCs treatment on myocardial tissue protein expression, Western blots were probed with antibodies against GLUT1, GLUT4, PFK, GAPDH, p70s6k (all from Abcam, Cambridge, UK) and β-actin (CWBIO Inc. China). Immunoreactive bands were visualized with chemiluminescence, and quantified with an image analyzer GS700 Bio-Rad densitometer and QuantityOne analysis software (Bio-Rad, Hercules, CA, USA). Target signals were normalized against β-actin and analyzed semiquantitatively using the NIH Image system.

### Immunohistochemical staining

For histological analysis, cardiac tissue samples were taken from the region of the left ventricle around the points of MSCs injection and cut into 5-μm thick sections. Sections were used for immunohistochemical staining with GLUT1 (Abcam), GLUT4 (Abcam), PFK (Santa Cruz), GAPDH (Abcam) and p70s6k (Santa Cruz) antibodies, respectively. The antibodies were labeled with biotin-conjugated secondary antibodies and protein expression was detected by the color reaction with diaminobenzidine (DAB) Substrate Kit (BD Pharmingen). Cells with brown granular DAB reaction product in the cytoplasm were considered positive for the protein.

### Statistical analysis

Statistical analysis was performed with SPSS 17.0 software for Windows (SPSS, Inc., Chicago, Illinois, USA). All data are presented as mean ± SD. After data’s normality test and homogeneity test of variances, nonparametric Mann-Whitney test was used for comparison between groups, and nonparametric Wilcoxon test was used for comparison between the baseline and the end. A value of *P* < 0.05 was considered significant.

## Additional Information

**How to cite this article**: Cai, M. *et al*. Bone Marrow Mesenchymal Stem Cells (BM-MSCs) Improve Heart Function in Swine Myocardial Infarction Model through Paracrine Effects. *Sci. Rep.* **6**, 28250; doi: 10.1038/srep28250 (2016).

## Supplementary Material

Supplementary Information
