# Erratum: Bone Marrow Mesenchymal Stem Cells (BM-MSCs) Improve Heart Function in Swine Myocardial Infarction Model through Paracrine Effects

**DOI:** 10.1038/srep31528

**Published:** 2016-09-02

**Authors:** Min Cai, Rui Shen, Lei Song, Minjie Lu, Jianguang Wang, Shihua Zhao, Yue Tang, Xianmin Meng, Zongjin Li, Zuo-Xiang He

Scientific Reports 6: Article number: 2825010.1038/srep28250; published online: 06 20 2016; updated: 09 02 2016

In this Article, Min Cai is incorrectly affiliated with ‘Department of Radiology, State Key Laboratory of Cardiovascular Disease, Fu Wai Hospital, National Center of Cardiovascular Disease, Peking Union Medical College & Chinese Academy of Medical Sciences, Beijing, China.’ In addition, Shihua Zhao is incorrectly affiliated with ‘Department of Nuclear Medicine, Shanxi Provincial People’s Hospital, Taiyuan, China.’ The correct affiliations are listed below:

Min Cai

Department of Nuclear Medicine, State Key Laboratory of Cardiovascular Disease, Fu Wai Hospital, National Center of Cardiovascular Disease, Peking Union Medical College & Chinese Academy of Medical Sciences, Beijing, China.

Department of Nuclear Medicine, Shanxi Provincial People’s Hospital, Taiyuan, China.

Shihua Zhao

Department of Radiology, State Key Laboratory of Cardiovascular Disease, Fu Wai Hospital, National Center of Cardiovascular Disease, Peking Union Medical College & Chinese Academy of Medical Sciences, Beijing, China.

