# Supplementary material for: Bone Marrow Mesenchymal Stem Cells (BM-MSCs) Improve Heart Function in Swine Myocardial Infarction Model through Paracrine Effects
Source: Sci Rep. 2016 Jun 20;6:28250. doi: 10.1038/srep28250 (PMC4913323; doi:10.1038/srep28250)
Supplement: Supplementary Information [file srep28250-s1.pdf]

Bone Marrow Mesenchymal Stem Cells (BM-MSCs) Improve Heart Function in Swine Myocardial Infarction Model through Paracrine Effects

Min Cai, Rui Shen, Lei Song, Minjie Lu, Jianguang Wang, Shihua Zhao, Yue Tang, Xianmin Meng, Zongjin Li, Zuo-Xiang He

Supplemental Figure Legend

Fig. S1 Phenotype identification of cultured BM-MSCs. BM-MSCs characterized by flow cytometry were positive for CD44, CD90, and negative for CD34, CD45 and HLA-DR.

Fig. S2 AHA left ventricular 17 segments diagram

Fig. S3 PET QPS software results analysis diagram

Fig. S4 Histology analysis of cardiac tissue sections from the BM-MSCs injection regions. Representative photographs of DAPI-positive nuclei of transplanted BM-MSCs at week 1 and week 4.

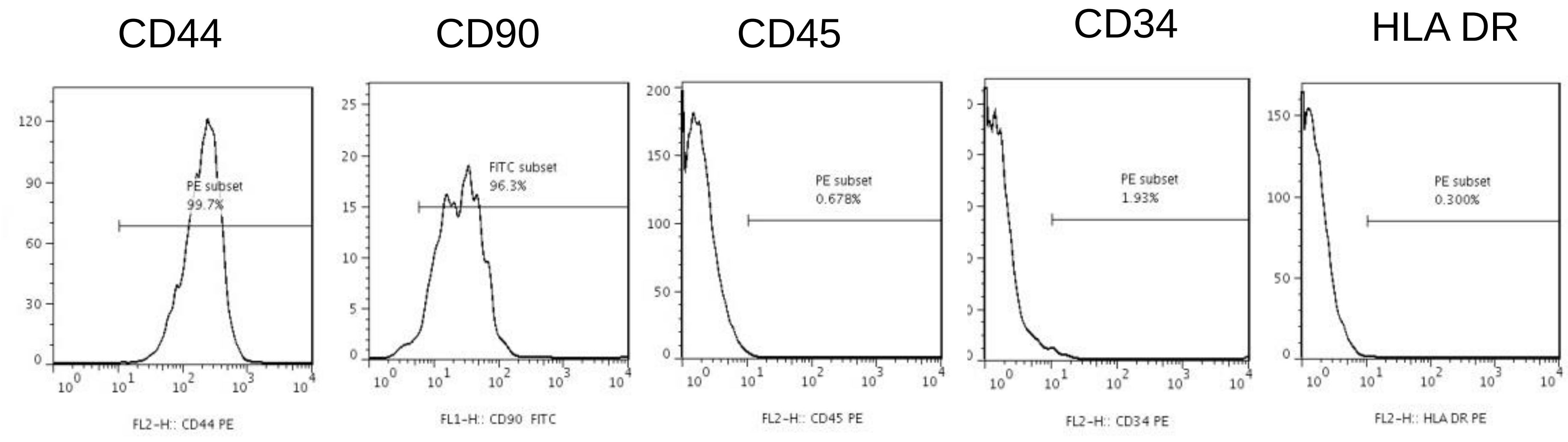

Supplemental Figure 1

Left Ventricular Segmentation

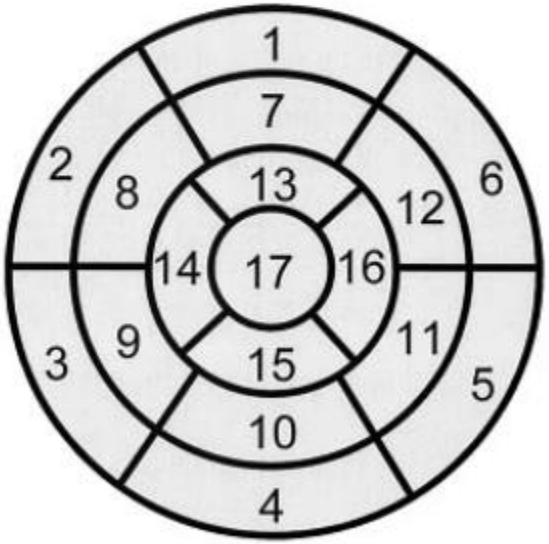

1. Basal anterior
2. Basal anteroseptal
3. Basal inferoseptal
4. Basal inferior
5. Basal inferolateral
6. Basal anterolateral
7. Mid anterior
8. Mid anteroseptal
9. Mid inferoseptal
10. Mid inferior
11. Mid inferolateral
12. Mid anterolateral
13. Apical anterior
14. Apical septal
15. Apical inferior
16. Apical lateral
17. Apex

Supplemental Figure 2

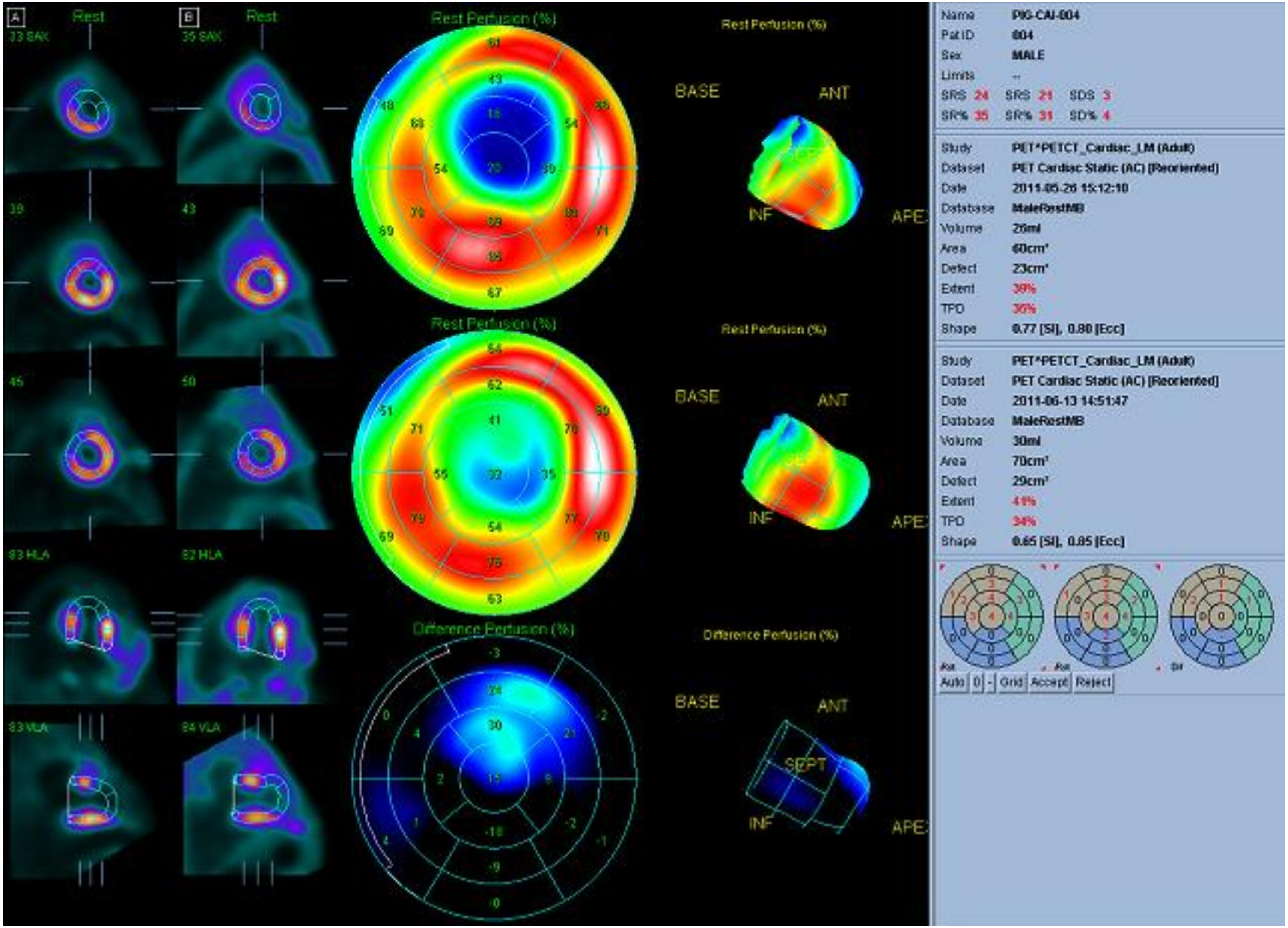

Supplemental Figure 3

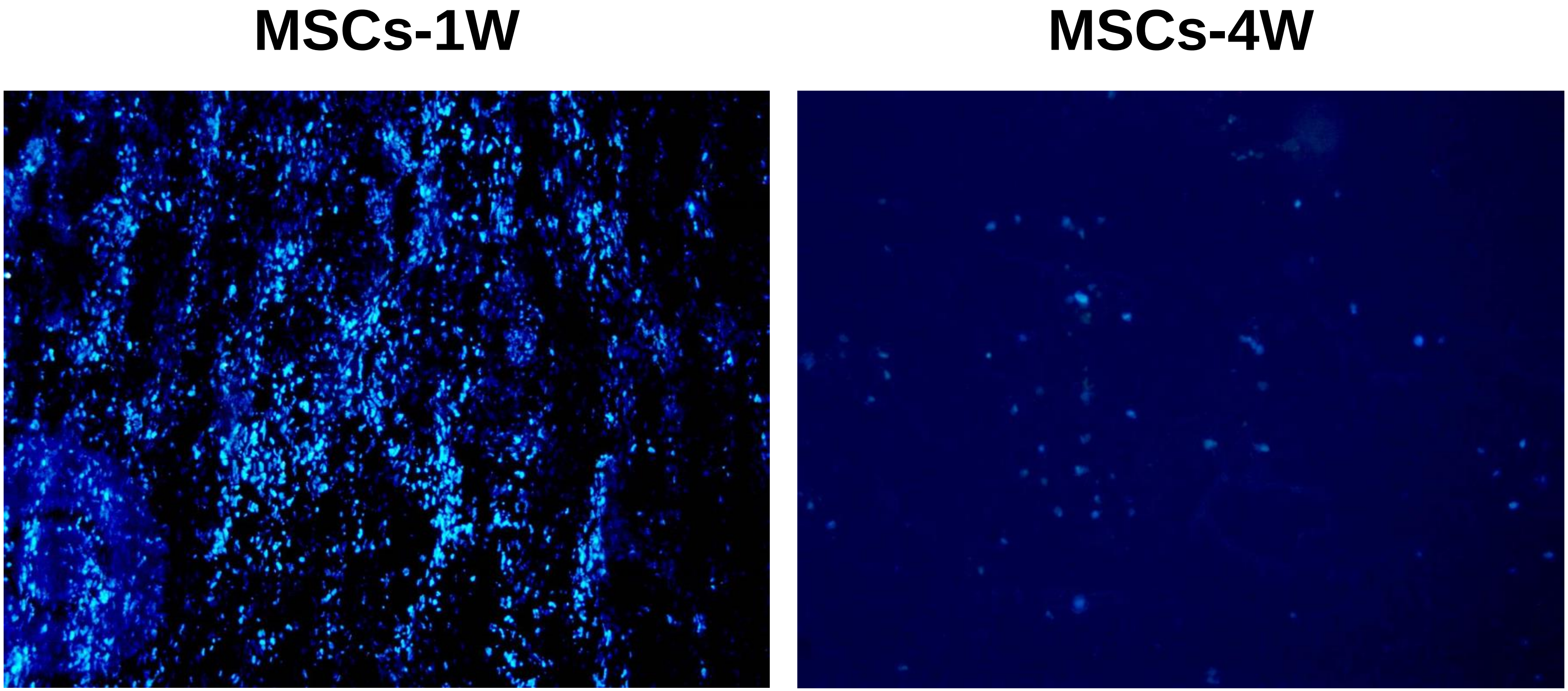

Supplemental Figure 4

Supplemental Table 1. Primers Used in This Study

Gene symbol	Primer sequence (5’-3’)	Product size (bp)
GLUT1	F: CCGCTTCCTGCTCATCAACC	138bp
	R: ACCTTCTTCTCCCGCATCATCT	
GLUT4	F: AGTATGTTGCGGATGCTATGGG	103 bp
	R: CCTCGGGTTTCAGGCACTT	
PFK	F: TGTTGAACGACCTCCAGAAAGC	119 bp
	R: CATATCGGTGCCGCAAAAGTC	
GAPDH	F: CTCCCCGTTCGACAGACAG	90 bp
	R: GCCAAATCCGTTCACTCCGAC	
PI3K	F: GCTGGTCCAGGCTGTGAAAT	113 bp
	R: TCAAGAACCAAAACAAGAAGTGACC	
Akt	F: GATCATGCAGCACCGTTTCTTC	112 bp
	R: GAAATACCTGGTGTCCGTCTCG	
mTOR	F: CCAACAAGATCCTGAAGAACATGT	150 bp
	R: CCCCGAAGTACAAGCGAGAC	
4E-BP1	F: TGC AGC CAG ACC CCA AGC CG	300bp
	R: CCG CTT ATC TTC TGG GCT ATT G	
p70s6k	F: AGG AGG CGG GAC GGC TTT TAC C	586bp
	R: CCA AGT AAA AGC AGG CAG TGT C	
β-Actin	F: TGAGATTGGCATGGCTTTATTTGG	122 bp
	R: GTCACCTTCACCGTTCCAGTTT	

After an initial denaturation at 95 ℃ for 10 min amplification was performed with 40 cycles of 95 ℃ for 15 s, 60 ℃ for 60 s.

Supplemental Table 2. Myocardial metabolism assessment of PET at week 4

	Control (n=10)			MSCs (n=10)			
	1 week	4 week	difference	1 week	4 week	difference	*P*
Minimum MSI	35.70±3.02	37.40±2.28	1.70±2.00	22.10±3.18	34.00±4.25	11.90±2.93	0.012
Summed MSI	1084.00±21.15	1089.90±24.47	5.90±27.98	1013.50±29.37	1075.50±28.30	62.00±23.30	0.005
SRS	20.90±2.11	19.80±1.37	-1.10±1.57	23.80±1.58	20.20±2.24	-0.36±1.53	0.287
SRS（%）	30.60±3.09	29.00±2.05	-1.60±2.28	35.10±2.34	29.80±3.31	-5.30±2.23	0.305
Defect（cm2）	25.50±2.60	29.50±3.31	4.00±1.90	28.90±2.48	27.20±4.06	-1.70±2.42	0.081
Extent（%）	38.90±3.67	39.90±2.64	1.00±2.09	42.40±2.46	37.70±3.79	-4.70±2.60	0.119
TPD（%）	31.50±2.83	31.80±2.12	0.30±1.37	36.00±2.57	31.70±3.52	-4.30±2.57	0.172
Abnormal segments	6.00±0.60	5.60±0.72	-0.40±0.75	6.90±0.50	5.70±0.79	-1.20±0.80	0.421
Summed MSI in abnormal segments	314.80±30.50	337.70±39.55	22.90±18.52	323.60±18.99	384.60±37.13	61.00±21.24	0.226
Mean MSI in abnormal segments	52.74±1.66	55.70±2.20	2.95±2.60	48.14±2.71	56.25±3.54	8.12±2.25	0.364

P value, Comparison of the difference between the two groups.

Supplemental Table 3. Control-4W Group and MSCs-4W Group regional myocardial metabolism assessment of PET

	Control-4W Group（n=10）			MSCs-4W Group（n=10）			
	1 week	4 week	difference	1 week	4 week	difference	*P*
apical-anterior	40.10±3.94	44.50±3.36	4.60±4.98	32.00±5.35	44.10±5.90	14.80±4.10	0.028
mid-anterior	64.80±3.33	70.00±4.62	5.60±3.75	57.40±4.00	65.30±4.66	9.30±2.91	0.545
apical-septal	55.50±3.21	59.40±2.53	4.40±3.71	52.00±2.55	61.60±2.67	12.49±3.95	0.289
mid-anteroseptal	64.10±2.75	68.10±3.29	4.30±3.03	62.80±2.85	69.50±2.17	7.10±2.51	0.623

P value, The comparison of the difference between the two groups.

Supplemental Table 4. Control-4W Group and MSCs-4 W Group cardiac function assessment

	Control-4W Group（n=10）			MSCs-4 W Group（n=10）			P
	1 week	4 week	difference	1 week	4 week	difference	
LVEF（%）	48.72±2.33	49.20±3.13	0.47±2.13	47.54±2.43	54.41±2.62	6.87±1.48	0.023
EDV（ml）	50.00±4.82	53.26±4.58	3.26±3.95	53.57±1.66	52.22±2.44	-1.35±1.48	0.496
ESV（ml）	25.67±2.79	26.73±2.51	1.06±1.96	27.07±1.67	22.85±1.91	-4.22±0.97	0.009
SV（ml）	24.33±2.49	26.51±2.99	2.18±2.35	26.52±1.46	29.35±1.84	2.83±1.11	0.650
CO（L/min）	1.73±0.17	1.95±0.23	0.22±0.27	1.96±0.13	2.23±0.14	0.27±0.10	0.472
CI（L/min·m^2）	2.19±0.21	2.37±0.26	0.18±0.32	2.48±0.18	2.83±0.14*	0.36±0.14	0.325
LVMASS-ED（g）	29.08±1.95	29.27±2.03	0.19±1.52	28.36±2.14	27.05±2.02	-1.31±1.74	0.631

LVEF, left ventricular ejection fraction; EDV, end-diastolic volume; ESV, end-systolic volume; SV, stroke volume; CO, cardiac output; CI, cardiac index; LVMASS-ED, left ventricular mass- end diastolic.

* The difference of MSCs-4W Group and Control-4W Group at the 4th week, are statistically significant, P <0.05

P value, The comparison of the difference between the two groups
